# 

**Published:** 1874-10

**Authors:** 


### Table of Contents.

### ORIGINAL COMMUNICATIONS.

Deportment of Blood-Clot and Blood-Deposits in Vessels and Tissues. *By Frank A. Ramsey, M.D., Knoxville, Tenn* ........ 385

Note on Croton-Chloral-Hydrate. *By I. Ott, M.D., Easton, Pa.*..... 400

Belladonna in Opium Poisoning. *By James O. Lewis, M.D., Greenwood, Fla*........ 403

How to get a Gold or Brass Ring from the Finger. *By William Hauser, M.D., Bartow, Ga.*........ 404

Crab-Orchard Salts. *By Luke P. Blackburn, M.D.*........ 405

On Nelaton's Method of Resuscitation from Chloroform Narcosis. *By J. Marion Sims, M.D., New York*........ 407

Thirty-Seven Cases of Urinary Calculus, *Treated by W. F. Westmoreland, M.D., Atlanta, Ga.*........ 415

My Experience with the Bi-Lateral Operation for Stone in the Bladder. *By F. A. Stanford, M.D., Columbus, Ga.*........ 425

### OUR EXCHANGES.

Self-Retaining Vaginal Speculum........ 428
Helmholtz's Treatment in Hay-Fever........ 431
Fissure of the Anus........
Treatment of Inverted Toe-nail........ 432
Carbozotate of Ammonia........ 433
Medical Maxims........
Milk in Febrile Diseases........ 434
Dangerous Burning Oils........ 435
Moonshine as Medicine........ 436
Maladies Produced by Boots and Shoes........ 438
Treatment of Cystic Goitre by Injection........ 440
Physiology of the Brain........
Aconite in Headache........ 442
Sterility, Result of Lithotomy........
Quinine Pills........
Pepsin in Diphtheria........ 443

### BIBLIOGRAPHICAL.

Transactions Alabama Medical Association........ 444

Jacobi on Infant Diet........ 446

Pamphlets........ 447

EDITORIAL........ 448

